# Role of Fiber in Symptomatic Uncomplicated Diverticular Disease: A Systematic Review

**DOI:** 10.3390/nu9020161

**Published:** 2017-02-20

**Authors:** Marilia Carabotti, Bruno Annibale, Carola Severi, Edith Lahner

**Affiliations:** 1 Medical-Surgical Department of Clinical Sciences and Translational Medicine, University Sapienza, Via di Grottarossa 1035, 00189 Rome, Italy; bruno.annibale@uniroma1.it (B.A.); edith.lahner@uniroma1.it (E.L.); 2 Department of Internal Medicine and Medical Specialties, University Sapienza, Viale del Policlinico 155, 00161 Rome, Italy; carola.severi@uniroma1.it

**Keywords:** diverticular disease, dietary fiber, supplemental fiber, symptomatic uncomplicated diverticular disease

## Abstract

Symptomatic uncomplicated diverticular disease (SUDD) is a syndrome characterized by recurrent abdominal symptoms in patients with colonic diverticula. There is some evidence that a high-fiber diet or supplemental fibers may reduce symptoms in SUDD patients and a high-fiber diet is commonly suggested for these patients. This systematic review aims to update the evidence on the efficacy of fiber treatment in SUDD, in terms of a reduction in symptoms and the prevention of acute diverticulitis. According to PRISMA, we identified studies on SUDD patients treated with fibers (PubMed and Scopus). The quality of these studies was evaluated by the Jadad scale. The main outcome measures were a reduction of abdominal symptoms and the prevention of acute diverticulitis. Nineteen studies were included, nine with dietary fiber and 10 with supplemental fiber, with a high heterogeneity concerning the quantity and quality of fibers employed. Single studies suggest that fibers, both dietary and supplemental, could be beneficial in SUDD, even if the quality is very low, with just one study yielding an optimal score. The presence of substantial methodological limitations, the heterogeneity of the therapeutic regimens employed, and the lack of ad hoc designed studies, did not permit a summary of the outcome measure. Thus, the benefit of dietary or supplemental fiber in SUDD patients still needs to be established.

## 1. Introduction 

Colonic diverticula are common in Western countries, affecting up to 60% of subjects over 70 years of age [1]. In about 80% of patients, colonic diverticula remain asymptomatic (diverticulosis), while approximately 20% of patients may develop abdominal symptoms (symptomatic uncomplicated diverticular disease, SUDD) and, eventually, complications such as bouts of diverticulitis or bleeding [2]. SUDD has been defined as a syndrome which is characterized by recurrent abdominal symptoms (i.e., abdominal pain and bloating resembling or overlapping those present in irritable bowel syndrome), attributed to diverticula in the absence of macroscopically evident alterations, other than the presence of diverticula [3,4]. The impact of these complaints is variable, and the severity and frequency of symptoms may range from mild and rare episodes, to a severe, chronic, recurrent disorder, impacting daily activities and the quality of life of patients [5,6]. About 4% of patients develop acute diverticulitis, an inflammatory process that may result in complications in about 15% of patients, with the development of abscesses, perforation, fistula, obstruction, or peritonitis [7]. A recurrence of diverticulitis after the first episode has been reported to occur in 15%–30% of patients [8,9].

The main goals of managing SUDD are both the reduction of abdominal symptoms and the prevention of acute diverticulitis. Even if recommendations for the treatment of SUDD have been issued by the medical societies of various countries [3,10,11,12,13], a standard therapeutic approach still remains to be defined. Fibers have been suggested for the treatment of SUDD patients, but the therapeutic benefit is not yet fully understood. Fibers might confer benefits by increasing fecal mass and regularizing bowel movements, as well as acting as prebiotics in the colon, favoring health-promoting species of the intestinal microbiota [14]. Fibers are defined as the edible parts of plants or the analogous carbohydrates that are resistant to digestion and absorption in the human small intestine, with complete or partial fermentation in the colon [15]. Fiber intake may be achieved by consuming fruits, vegetables, and cereal grains (dietary fibers), and/or by diet supplementation with specific commercial preparations containing fibers (supplemental fibers). 

A previous systematic review assessed whether a high-fiber diet can improve symptoms or prevent complications of diverticular disease. Few studies were identified, and the authors concluded that evidence for a therapeutic benefit of a high-fiber diet in the treatment of diverticular disease is poor [16]. 

This systematic review aims to update the evidence on the efficacy of treatment with fiber in SUDD, in terms of the reduction of symptoms and the prevention of acute diverticulitis. 

## 2. Methods

### 2.1. Study Selection

The search was conducted according to the PRISMA (Preferred Reporting Items for Systematic Reviews and Meta-Analyses) guidelines [17]. The electronic databases PubMed MEDLINE (U.S. National Library of Medicine, Bethesda, MD, USA) and Scopus were systematically searched according to the following search strategy, using the following MesH terms:

(((“diverticulum” [MeSH Terms] OR “diverticulum” [All Fields] OR “diverticulosis” [All Fields]) OR diverticular [All Fields] OR (“diverticulum” [MeSH Terms] OR “diverticulum” [All Fields] OR “diverticula” [All Fields]) OR (“diverticulitis” [MeSH Terms] OR “diverticulitis” [All Fields])) AND ((“colon” [MeSH Terms] OR “colon” [All Fields]) OR (“colon” [MeSH Terms] OR “colon” [All Fields] OR “colonic” [All Fields]) OR (“colon, sigmoid” [MeSH Terms] OR (“colon” [All Fields] AND “sigmoid” [All Fields]) OR “sigmoid colon” [All Fields] OR “sigmoid” [All Fields])) AND ((“dietary fiber” [MeSH Terms] OR (“dietary” [All Fields] AND “fiber” [All Fields]) OR “dietary fiber” [All Fields] OR “fiber” [All Fields]) OR fibre [All Fields] OR (“diet” [MeSH Terms] OR “diet” [All Fields] OR “dietary” [All Fields]) OR insoluble [All Fields] OR soluble [All Fields] OR (“fruit” [MeSH Terms] OR “fruit” [All Fields]) OR (“vegetables” [MeSH Terms] OR “vegetables” [All Fields] OR “vegetable” [All Fields]) OR (“(1-6)-alpha-glucomannan” [Supplement *] OR “(1-6)-alpha-glucomannan” [All Fields] OR “glucomannan” [All Fields]) OR (“starch” [MeSH Terms] OR “starch” [All Fields]) OR fructooligosaccharides [All Fields] OR bran [All Fields] OR (“inulin” [MeSH Terms] OR “inulin” [All Fields]) OR (“psyllium” [MeSH Terms] OR “psyllium” [All Fields]))) AND (“humans” [MeSH Terms] AND (English [lang] OR French [lang] OR German [lang] OR Italian [lang] OR Spanish [lang]) AND “adult” [MeSH Terms]) AND (“therapy” [Subheading] OR “therapy” [All Fields] OR “treatment” [All Fields] OR “therapeutics” [MeSH Terms] OR “therapeutics” [All Fields]) AND (“humans” [MeSH Terms] AND (English [lang] OR French [lang] OR German [lang] OR Italian [lang] OR Spanish [lang]) AND “adult” [MeSH Terms]).

The search strategy excluded reviews, meta-analyses, case reports, and animal studies. The following study types were included: randomized controlled trials (blinded and/or placebo-controlled), open randomized clinical trials, and non-randomized open studies. Pediatric subjects were excluded from this review. No publication data restriction was imposed. Reports published in English, German, French, Italian, and Spanish were considered. 

Clinical studies published up to 7 October 2016 were considered for inclusion in this review, if they described in adults (>18 years) with SUDD, the efficacy of fiber treatment with respect to the baseline (i) on reduction or remission of abdominal symptoms; and/or (ii) on prevention of acute diverticulitis.

Potentially relevant articles were independently screened for eligibility in an un-blinded standardized manner by the two reviewers (M.C., E.L.), initially by abstract, and then by full text when necessary, in order to determine whether they met the inclusion criteria. Reviews, letters, books, and/or editorials were excluded on the basis of the abstract and/or title; in other cases, the judgement of inclusion/exclusion was based on an evaluation of the full-text. Disagreement between reviewers was resolved by discussion. The reference lists of the identified articles, as well as of the identified relevant reviews, were manually searched for additional studies that may have been overlooked using a computer-assisted search strategy.

### 2.2. Data Extraction 

We developed a data extraction sheet, pilot-tested it on three randomly-selected included studies, and refined it accordingly. One review author (M.C.) extracted the data from the included studies and the second author (E.L.) checked the extracted data. Disagreements were resolved by discussion between the two review authors. The following information was extracted from each included paper: (1) author and year of publication; (2) characteristics of fibers; (3) characteristics of study participants (number, mean age, and gender); (4) diagnosis of SUDD; (5) study type and treatment arms; (6) type of intervention; (7) follow-up; (8) outcome measure (reduction of abdominal symptoms; occurrence of acute diverticulitis); (9) efficacy of intervention; (10) adverse effects of fiber arms; (11) single or multiple centers. 

The diagnosis of SUDD was considered appropriate when patients with colonic diverticula had recurrent abdominal symptoms such as abdominal pain, which were eventually associated with bloating or bowel habit alteration [3]. Studies which did not completely fulfill this definition were not excluded a priori, but the specific clinical settings were singularly extracted and described in detail. 

For the purpose of this paper, dietary fibers were defined as the intake of food fibers in fruits, vegetables, and cereal grains. A high-fiber diet has been defined as at least a 30 g daily intake of dietary fibers [18]. When indicated, the amount of daily fiber intake was extracted from each paper. Supplemental fibers were defined as diet supplementation with specific commercial preparations containing one or more types of fiber. 

### 2.3. Statistical Analysis 

Originally, a meta-analysis was planned in order to provide a numerical estimate of the overall effect of interest, for which the outcome measure (effect size) comprised the proportion of patients who showed a positive response to fiber treatment with respect to the baseline, or with respect to controls, defined as the reduction of abdominal symptoms and prevention of acute diverticulitis. Due to the heterogeneity of the retrieved studies and their low quality, a meta-analysis was not considered applicable. The efficacy of the interventions reported in the retrieved studies was described in a qualitative manner.

### 2.4. Quality Assessment

The two reviewers evaluated the quality of all of the included studies, using the Jadad scale for randomized controlled trials [19]. This scale awards a maximum of five points to each study. The considered categories are randomization, blinding of outcome assessment, description of withdrawals and dropouts, and description and appropriateness of randomization and blinding. A study can be awarded a maximum of one point for each category (Table S1). Discrepancies in the quality assessment were discussed and resolved by the two reviewers. 

## 3. Results 

### 3.1. Search Results 

**Figure 1:**
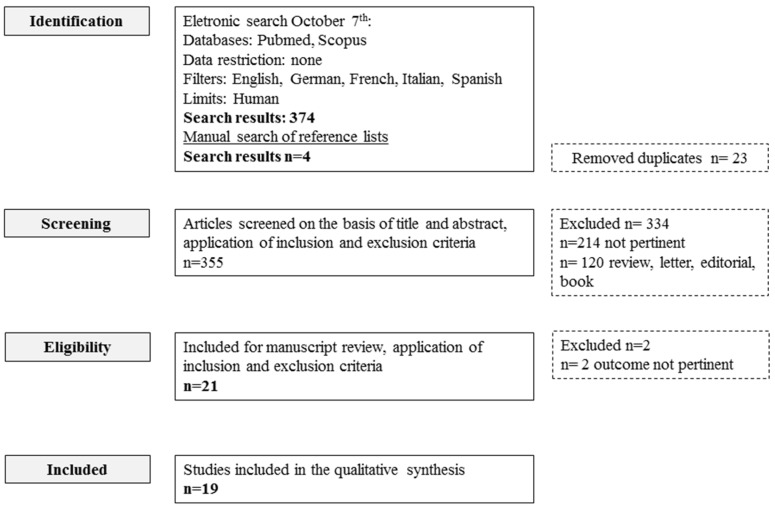
Flow-chart of study selection.

The electronic search study identified a total of 374 records from electronic databases, 351 of which were unique (Figure 1). 

Manual searching of a reference list of potentially relevant papers contributed another four articles. The articles were screened on the basis of the title and abstract and, after application of the inclusion and exclusion criteria, 21 articles were retrieved for a full-paper evaluation. Of these 21 papers, 19 met the eligibility criteria and were subjected to data extraction. Two studies were excluded because the outcome was not pertinent to the present study purpose, since it only evaluated constipated patients [20,21]. Thus, 19 articles were included for qualitative synthesis (Table 1).

### 3.2. Quality Assessment

Details of the quality assessment of the included studies are given in Supplementary Table S1 (see Supplementary material). Of the 19 studies included, six achieved 0 points [26,27,35,36,39,40], three achieved 1 point [25,29,30], two achieved 2 points [28,38], seven achieved 3 points [22,23,24,31,33,34,36], and only one achieved 5 points [33], according to Jadad scale. 

### 3.3. Characteristics of Included Studies

The main characteristics of the 19 included studies are summarized in Table 2. Considering the high heterogeneity of the fibers used, studies are grouped on the basis of dietary (nine articles) or supplemental fibers (ten articles).

In thirteen studies, SUDD was appropriately diagnosed [22,23,24,25,26,28,30,32,33,34,36,38,39], while in six studies, the diagnosis was not completely appropriated [27,29,35,37,40] (see Table 2). The latter studies include three in which SUDD patients with diverticulosis were included [29,35,37], and in the other two studies, SUDD patients with acute diverticulitis were included [27,28,29,30,31,32,33,34,35,36,37,38,39,40]. In one study, patients who had reported a recent episode of colonic diverticulitis, but were currently in remission, were included [31].

### 3.4. Dietary Fiber

Articles concerning dietary fibers were performed over a period of 40 years, from 1972 to 2012, and only three of them were published in the last 10 years [22,23,24]. Six of these were single center studies and three were multicenter studies [22,23,24]. Six studies were conducted in the United Kingdom [25,26,27,28,29,30] and three were completed in Italy [22,23,24]. 

An overall number of 736 patients with SUDD were investigated, for which the female gender was slightly prevalent (*n* = 391), but in two articles [26,27,28,29], the gender of the patients was lacking. Patients had a mean age of 64 years, ranging from 59 to 71 years. In one study, the age of patients was lacking [26].

With regard to the study type, four were randomized controlled open trials [22,23,24,25], two were retrospective studies [26,27], one was a double-blind RCT [28], one was a prospective partly cross-over study [29], and the remaining one was an un-controlled study [30].

With regard to the fibers used, in the majority of the studies, patients were treated with dietary fibers [22,23,24,25,26,27], in two articles crispbread was used [28,29], and in the last study, a high residue, low sugar with unprocessed bran was utilized [30]. In addition, the amount of dietary fiber utilized was variable, ranging from 20 [24] to 96 gr/day [29]. In five studies, a high-fiber diet was employed [22,23,25,27,29], but in the other studies, the dosage of fiber seemed to be lower than 30 gr daily [24,26,28,30]. Unfortunately, it was not possible to assess the proportion of soluble or insoluble fibers for each dietary regimen, since its exact composition was not reported. 

The follow-up protocol was very variable between studies, ranging from three [25,26,27,28] to 65 months [27]. Also, the interventions were variable between studies: in four studies, the dietary fiber was a control arm and was compared in two articles to symbiotic preparations [22,23], in another it was compared to rifaximin [24], and in the last it was compared to lactulose [25]. In one study, a high-fiber diet was compared to one which was not high in fiber [26], and in two studies, high-fiber crispbread was compared to lower fiber crispbread [28,29]. One study used a high-fiber diet without a control arm [27], and the other study used a high-residue, low sugar diet with unprocessed bran [30]. With regard to the outcome measures, seven articles assessed the reduction of abdominal symptoms [22,23,25,27,28,29,30], and two assessed the reduction of symptoms and/or complications. 

Two of the most recent open RCT studies compared a high-fiber diet with the combined treatment of a high-fiber diet and a symbiotic preparation [22,23]. In the first study, both treatments significantly reduced abdominal pain [22], whereas in the second, the high-fiber diet alone did not improve abdominal symptoms, compared to the baseline [23]. Another open RCT study compared a high-fiber diet with the combination of a high-fiber diet and rifaximin, and showed that both treatments significantly improved abdominal symptoms, compared to the baseline [24]. The occurrence of diverticulitis was reduced during the administration of a high-fiber diet in comparison to one which was not high in fiber, at a follow-up of 65 months [26]. Another study showed a similar frequency of diverticulitis occurrence in both treatment arms, for both dietary fiber and dietary fiber plus rifaximin, after 24 months [24]. Table 3 summarizes the type of intervention, follow-up protocols, the outcome measure, and the efficacy of each intervention included in the selected studies.

Due to the poor quality of the studies and the heterogeneity of the study design (mean Jadad score 1.5 ± 1.2 points), a meta-analysis could not be performed to provide a pooled estimate of the outcome measure. With regard to adverse effects, data were not reported in four studies [26,27,28,29], no adverse effect was observed in two studies [22,23,24,25], and in three studies, some minor effects were reported [23,24,30].

### 3.5. Supplemental Fiber 

These studies were performed over a range of 37 years, from 1976 to 2013, and just one article was published in the last 10 years. Five studies were single center in nature [35,37,38,39,40] and five were multicenter studies [31,32,33,34,36]. Five studies were conducted in the United Kingdom [35,36,37,39,40], three in Italy [32,33,34], one in Spain [31], and one in the USA [38]. The ten included studies investigated an overall total number of 1707 patients, of which 830 were female, but in three studies, the gender of the patients was lacking [37,38,39]. Patients had a median age of 62 years, ranging from 54 to 66 years, but in two studies, the age of patients was lacking [39,40].

With regard to the study type, three were open randomized controlled trials [31,32,34], two were double blind-cross over studies [36,38], one was a double-blind randomized placebo controlled study [33], two were open un-controlled studies [35,39], and the last was a cross-over RCT [40]. 

With regard to the type of supplementation, patients were treated with glucomannan [32,34], ispaghula [35,36,37], bran [36,37,38,40], plantago ovata [31], and methylcellulose [38]. None of the studies achieved a high dosage of fiber intake with the prescribed supplementation regimen. Because the fiber intake of the diet was not reported, it may be that the total amount of the daily fiber intake is higher than that reported. With regard to fiber solubility, soluble fibers were used in five studies [31,32,33,34,35], both insoluble and soluble fibers were used in two studies [36,37], and in three studies, insoluble fibers were used [38,39,40]. 

With regard to the follow-up protocol, the studies were variable, ranging from one to 12 months. Five studies observed patients for a period of less than six months [35,36,37,38,40]. In addition, the interventions were very variable: in four studies, fibers were administrated together with a drug (rifaximin) and compared with the efficacy of the fiber alone [32,33,34]; in two studies, the efficacy of ispaghula and bran was respectively compared to a placebo [36] or lactulose [37], or were administrated as a unique arm of treatment in open un-controlled studies [35,39]. In another study, the efficacy of bran was compared to a high roughage diet or bulk laxative [40], or methylcellulose was compared to a placebo [38]. With regard to the outcome measures, the majority of studies evaluated the reduction of abdominal symptoms [33,35,36,37,38,39,40], two evaluated the reduction of symptoms and the occurrence of diverticulitis [32,34], and another study only considered the recurrence of diverticulitis [31]. In three open RCTs, the efficacy of glucomannan (2 or 4 gr/day) was compared to glucomannan, together with cyclic rifaximin, analysing the improvement of abdominal symptoms in SUDD patients [32,33,34]. In all three studies, a significant reduction of abdominal symptoms in the treatment arm with just glucomannan was achieved. In two of these three studies, the glucomannan treatment arm had a similar occurrence of diverticulitis to the antibiotic arm [33,34], while in the Latella study, the glucomannan arm treatment reported more complications (*p* < 0.05) [32]. Table 4 summarizes the type of intervention, follow-up protocols, outcome measures, and efficacy of interventions. 

Also, due to the poor quality of the studies and their heterogeneity (mean Jadad score 1.9 ± 1.8 points), a meta-analysis could not be performed. 

On the basis of the heterogeneity of these papers, it was not possible to perform a sub-analysis assessing the differences between dietary and supplemental fibers, the effects of high- or low fiber diet/supplementation, or the differences between soluble and insoluble fibers. 

With regard to adverse effects, data were not reported in six studies [34,35,37,38,39,40], some minor adverse effects were reported in the fiber arm in three studies [31,32,36], and in another study, the only double-blind randomized placebo controlled report, no adverse effects were observed [33]. 

## 4. Discussion

Dietary fibers are defined as the edible parts of plants or the analogous carbohydrates that are resistant to digestion and absorption in the human small intestine, with complete or partial fermentation in the colon [15]. Dietary fibers include non-starch polysaccharides, resistant oligosaccharides, and other carbohydrates, such as resistant starch and dextrins, and lignin [41,42]. Non-starch polysaccharides can be further subdivided into soluble and insoluble fibers: soluble fibers dissolve in water-forming viscous gels, bypass the digestion of the small intestine, and are easily fermented by the gut microbiota (i.e., pectins, gums, inulin-type fructans, and some hemicelluloses). In contrast, insoluble fibers are not water soluble, they do not form gels due to their water insolubility, and fermentation is severely limited (i.e., lignin, cellulose, and some hemicelluloses). The Academy of Nutrition and Dietetics declared that the adequate intake of fiber is 14 gr total per 1000 kcal, or 25 gr for adult women and 38 gr for adult men [18]. In Western countries, the daily fiber intake can change from region to region, and may change over the years. The mean intake of dietary fiber in the United States is 17 gr/day, with only 5% of the general population meeting the adequate intake [18]. In contrast, in a recent study evaluating the changes in food consumption and nutrient intake in a Mediterranean cohort, it has been observed that fiber intake has a baseline of 24.3 ± 9.4 gr/day; after 10 years, it was observed that fiber intake increased by 1.8 gr/day, thus augmenting over time [43]. Even if the health benefits of dietary fibers have long been appreciated, especially for their effect on cardiovascular diseases, type II diabetes, glycemic control, and gastrointestinal conditions [14], these data underline that dietary habits in Western countries may be far from the recommended adequate intake.

With regard to the risk of developing colonic diverticula, it has been proposed that “fiber deficiency”, caused by the spreading of refined carbohydrates in the Western diet, may play an important role. However, little evidence is available to substantiate this hypothesis [44,45], and more recently, this concept has been reviewed. A colonoscopy-based, cross-sectional study on the dietary risk factors for diverticulosis found that a high fiber diet was even associated with a higher prevalence of colonic diverticula [46]. The association with dietary fiber intake was dose-dependent and stronger when limited to cases with multiple diverticula. Although it has been suggested that a high-fiber diet does not protect against the development of colonic diverticulosis, it may reduce the abdominal symptoms related to diverticular disease. 

Patients with SUDD may complain of chronic recurrent abdominal symptoms, and fibers might confer benefits by increasing fecal mass and promoting the regularity of bowel movements. Another beneficial effect of fibers in the treatment of SUDD may be ascribed to their capability to act as prebiotics in the colon, by favoring health-promoting species of the intestinal microbiota, especially bifidobacteria and lactobacilli [14]. 

Diverticular disease is a complex, multifactorial disorder, in which the gut microbiota could play a key role. Recently, Barbara et al. reported that patients with diverticular disease showed depletion of microbiota members with anti-inflammatory properties, including Clostridium cluster IV, Clostridium cluster IX, Fusobacterium, and Lactobacillaceae, with microbiota changes being correlated with mucosal immune activation [47]. The gut microbiota, indeed, shifts rapidly in response to dietary changes, particularly with fiber intake [48]. For this reason, high-fiber intake represents a promising treatment option in diverticular disease. In this condition, a high-fiber diet based on fruits, vegetables, and cereal grains may be difficult to achieve and should be supported with an adequate dietary counseling, and in a subset of patients, the use of supplemental fibers might be useful. 

The beneficial effect on potential microbiota changes achieved with dietary fibers, based on different amounts of soluble and insoluble fibers, or the supplementation of commercial fibers of the same type, may be different and not necessarily comparable. However, it was not possible to perform a sub-analysis assessing the differences between dietary or supplemental fibers, or between high- or low-fiber diet/supplementation. Unfortunately, on the basis of these studies, it was not possible to assess differences between the effect of soluble or insoluble fibers, even if the relative amount might have influenced the outcome of treatment. In patients with irritable bowel syndrome, a condition that might be considered similar to SUDD, the effect of fibers appears to be limited to the soluble type [49]. 

In clinical practice, a high-fiber diet or fiber supplementation are commonly used in patients affected by diverticular disease, even if most recommendations are based on poor evidence. A previous systematic review, performed in 2012, was restricted to the use of a high-fiber diet in diverticular disease and only included controlled studies in the English language, reporting that high-quality evidence for a high-fiber diet in the treatment of this condition is scarce [16]. 

The present systematic review represents an attempt to provide an updated measure of evidence on the efficacy of dietary and supplemental fibers in SUDD, in terms of the reduction of abdominal symptoms and the prevention of acute diverticulitis.

The research was updated until October 2016, considering randomized controlled trials (blinded and/or placebo-controlled), open randomized clinical trials, non-randomized open studies, and also taking into consideration papers in German, French, Italian, and Spanish. The selected studies, all of which came from Western countries with just one study from the USA, presented a high heterogeneity concerning the quantity and quality of the fibers employed, notwithstanding dietary and supplemental fibers, which were evaluated separately. However, the quality of the studies was very low, with just one study yielding an optimal score [33]. Based on the available studies, it was not possible to draw conclusions regarding the efficacy of fiber treatment in SUDD patients, neither in terms of the reduction of abdominal symptoms, nor for the prevention of acute diverticulitis. 

## 5. Conclusions

Single low quality studies suggest that fibers, both dietary and supplemental, could be beneficial in the treatment of SUDD. The presence of substantial methodological limitations, the heterogeneity of therapeutic regimens employed, and the lack of ad hoc designed studies, do not permit a summary of the outcome measures. Up to now, high-quality evidence on the efficacy of fiber treatment for the reduction of symptoms in SUDD and for the prevention of acute diverticulitis, is lacking. Thus, further, well-designed studies, specifically focusing on the efficacy of fibers in SUDD, dietary or supplemental, are needed. 

## Figures and Tables

**Table 1:** Type of fibers and dosage used in the included studies.

Author, Year (Reference)	Type of Fibers and Dosage
	Dietary
Lahner E., 2012 [22]	Dietary fiber (at least 30 gr/day)
Annibale B., 2011 [23]	Dietary fiber (at least 30 gr/day)
Colecchia A., 2007 [24]	Dietary fiber (at least 20 gr/day)
Smits B.J., 1990 [25]	Dietary fiber (30–40 gr/day)
Leahy A.L., 1985 [26]	Dietary fiber (>25 gr/day)
Hyland J.M.P., 1980 [27]	Dietary fiber (37.9 gr/day)
Brodribb A.J.M., 1977 [28]	Bran crispbread (6.7 gr/day) Wheat crispbread (0.6 gr/day)
Plumley P.F., 1973 [29]	High-fiber crispbread (96 gr/day total unavailable fraction)
Painter N.S., 1972 [30]	High-residue, low sugar diet with unprocessed bran (12–14 gr/day)
	Supplemental
Lanas A., 2013 [31]	Plantago ovata (7 gr/day)
Latella G., 2003 [32]	Glucomannan (4 gr/day)
Papi C., 1995 [33]	Glucomannan (2 gr/day)
Papi C., 1992 [34]	Glucomannan (2 gr/day)
Thorburn H.A., 1992 [35]	Ispaghula husk (7 gr/day)
Ornstein M.H., 1981 [36]	Bran (6.99 g/day) Ispaghula (9.04 gr/day)
Eastwood M.A., 1978 [37]	Bran (20 g/day) Isphaghula (2 sachets/day)
Hodgson W.J.B., 1977 [38]	Methylcellulose (1000 mg/day)
Brodribb A.J.M., 1976 [39]	Wheat bran (24 gr/day)
Taylor I., 1976 [40]	High roughage diet with bran supplement Bran (18 gr/day)

**Table 2:** Main characteristics of the 19 selected studies on fibers in symptomatic uncomplicated diverticular disease (SUDD).

Author, Year (Reference)	N/F/Mean Age	Diagnosis of SUDD	Study Type/Arms	Single Center Yes/No
Dietary				
Lahner E., 2012 [22]	44/35/66	App	Open RCT/2	No
Annibale B., 2011 [23]	50/32/65	App	Open RCT/3	No
Colecchia A., 2007 [24]	307/189/62	App	Open RCT/2	No
Smits B.J., 1990 [25]	43/28/59	App	Open RCT/2	Yes
Leahy A.L., 1985 [26]	56/-/-	App	Retrospective study/2	Yes
Hyland J.M.P., 1980 [27]	100/73/67	NcApp ^1^	Retrospective study/1	Yes
Brodribb A.J.M., 1977 [28]	18/9/60	App	Double-blind RCT	Yes
Plumley P.F., 1973 [29]	48/-/71	NcApp ^2^	Prospective interventional, partly cross-over study/2	Yes
Painter N.S., 1972 [30]	70/25/60	App	Prospective interventional study, not controlled/1	Yes
Supplemental				
Lanas A., 2013 [31]	165/59/54	NcApp ^3^	Open RCT/2	No
Latella G., 2003 [32]	968/501/63	App	Open RCT/2	No
Papi C., 1995 [33]	168/100/62	App	Double-blind placebo controlled/2	No
Papi C., 1992 [34]	217/112/65	App	Open RCT/2	No
Thorburn H.A., 1992 [35]	10/4/66	NcApp ^4^	Open/1	Yes
Ornstein M.H., 1981 [36]	58/36/64	App	Double-blind, cross-over, RCT/3	No
Eastwood M.A., 1978 [37]	31/-/60	NcApp ^5^	Prospective, not randomized/3	Yes
Hodgson W.J.B., 1977 [38]	30/18/60	App	Double-blind, cross-over RCT/2	Yes
Brodribb A.J.M., 1976 [39]	40/-/-	App	Prospective, not controlled/1	Yes
Taylor I., 1976 [40]	20/-/-	NcApp ^6^	Cross-over RCT	Yes

App: appropriate; NcApp: not completely appropriate; F: female gender; N: number of patients; RCT: randomized controlled trial; ^1^ an unspecified number of pts with acute diverticulitis were included; ^2^ 4 pts with diverticulosis were included; ^3^ pts with a recent episode of colonic diverticulitis, current in remission were included, ^4^ 3 pts with diverticulosis were included; ^5^ an unspecified number of pts with diverticulosis were included; ^6^ 8 pts with acute diverticulitis were included.

**Table 3:** Intervention and follow-up protocol in the selected studies of dietary fiber treatment in symptomatic uncomplicated diverticular disease (SUDD).

Author, Year (Reference)	Intervention	FU (Months)	Outcome Measure	Efficacy of Intervention	Adverse Effects
Lahner E., 2012 [22]	T1: High-fiber diet T2: Symbiotic preparation + high-fiber diet	6	Reduction of abdominal symptoms	T1 and T2: decrease of abdominal pain <24 h and >24 h in T1 and T2 (*p* < 0.05); T1 and T2: decrease of intensity of abdominal pain <24 h and bloating (*p* < 0.05)	None
Annibale B., 2011 [23]	T1: High-fiber diet T2: Symbiotic (1 sachet bid) + high-fiber diet for 14 days/months T3: Symbiotic (2 sachets bid) + high-fiber diet for 14 day/months	6	Reduction of abdominal symptoms	T1: no significant efficacy; T2 and T3: decrease of bloating VAS (*p* < 0.05), and abdominal pain >24 h (*p* = 0.016)	T3: 1 pt diarrhea
Colecchia A., 2007 [24]	T1: Dietary fiber T2: Dietary fiber + Rifaximin 400 mg bid for 7 days/months	24	Reduction of abdominal symptoms and diverticulitis	T1 and T2: significant reduction of symptomatic score compared to baseline; T1 has similar frequency of diverticulitis of T2 (4 pts in T1 vs. 2 pts in T2; *p* = 1)	Nausea, headache and weakness, T1: 3 pts, T2: 4 pts (*p* = ns)
Smits B.J., 1990 [25]	T1: High-fiber diet T2: Lactulose 15 mL bid	3	Reduction of abdominal symptoms	T1 and T2: reduction of abdominal pain frequency (T1: *p* = 0.022 and T2 *p* = 0.0015) and severity (T1: *p* = 0.028 and T2 *p* = 0.009) in comparison to baseline	T1: none, T2 :4 pts drops out for abdominal pain, nausea, vomiting
Leahy A.L., 1985 [26]	T1: High-fiber diet T2: Non High-fiber diet	65	Occurrence of symptoms and diverticulitis	T1 reported fewer symptoms and surgery or complications in comparison to T2 (19.3% vs. 44% and 6.4% vs. 32%; *p* < 0.05)	Not reported
Hyland J.M.P., 1980 [27]	T1: High dietary fiber	60	Reduction of abdominal symptoms	T1: 91% (91/100) were symptoms free at 5 years	Not reported
Brodribb A.J.M., 1977 [28]	T1: Bran crispbread T2: Wheat crispbread	3	Reduction of symptoms	T1 reduction of total symptom score in comparison to T2 (from 34.3 to 8.1 and from 42.0 to 35.1 respectively *p* < 0.002)	Not reported
Plumley P.F., 1973 [29]	T1: High fiber crispbread T2: Standard crispbread for at least 2 months	21	Reduction of abdominal symptoms	T1: 29/42 (69%) pts with pain were controlled satisfactory. Only 14 pts took part in the cross over trial (taking T2), but no results are available	Not reported
Painter N.S., 1972 [30]	T1: High-residue, low sugar diet with unprocessed bran	22	Reduction of abdominal symptoms	T1: 88.6% of symptoms relieved or resolved	8 pts did not tolerate bran diet

FU: follow-up; pts: patients; T1: treatment arm 1; T2: treatment arm 2; T3: treatment arm 3.

**Table 4:** Intervention and follow-up protocol in the selected studies of supplemental fiber treatment in symptomatic uncomplicated diverticular disease (SUDD).

Author, Year (Reference)	Intervention	FU (Months)	Outcome Measure	Efficacy of Intervention	Adverse Effects
Lanas A., 2013 [31]	T1: Plantago ovata 3.5 gr bid T2: Plantago ovata 3.5 gr bid + Rifaximin 400 mg bid for 7 days/months	12	Recurrence of diverticulitis	T1: recurrences in 17/88 pts (19.3%) T2: recurrences in 8/77 (10.4%) T1 had a significant higher risk of recurrence *p* = 0.025; OR 3.2 (95% CI: 1.16–8.82)	T1: 13/88 (14.8%) T2: 17/77 (22.1%) (*p* = 0.225)
Latella G., 2003 [32]	T1: Glucomannan 4 gr/day T2: Glucomannan 4 gr/day + Rifaximin 400 mg bid for 7 days/months	12	Reduction of abdominal symptoms and occurrence of diverticulitis	T1 and T2: significant reduction of global symptomatic score in comparison to baseline; T2 had more asymptomatic pts in comparison to T1: 29.2% vs. 56.5% pts at 12 months (*p* < 0.001); T1 reported more diverticulitis in comparison to T2: 11 pts vs. 6 pts (*p* < 0.05).	Nausea, headache, and asthenia: T1: 5 (1.34%) T2: 10 (1.68%) (*p* = 0.7932)
Papi C., 1995 [33]	T1: Glucomannan 2 gr/die + placebo T2: Glucomannan 2 gr/die + Rifaximin 400 mg bid for 7 days/month	12	Reduction of abdominal symptoms	T1 and T2: reduction of global symptom score in comparison to baseline; T2 had more asymptomatic pts in comparison to T1: 68.9% vs. 39.5% pts at 12 month (*p* = 0.001); No differences in preventing diverticulitis.	None
Papi C., 1992 [34]	T1: Glucomannan 2 gr/die T2: Glucomannan 2 gr/die + Rifaximin 400 mg bid for 7 days/months	12	Reduction of abdominal symptoms and occurrence of diverticulitis	T1 and T2: reduction of global symptom score in comparison to baseline. T2 had a marked reduction of score in comparison to T1 (47.6% vs. 63.9%: *p* < 0.001); No differences in preventing diverticulitis (T1: 3 vs. T2: 0, *p* = 0.2467)	Not reported
Thorburn H.A., 1992 [35]	T1: Ispaghula husk	1	Reduction of abdominal symptoms	Improvement of abdominal pain in 71.4% (5/7); Bowel habit improves in 66.6% (6/9)	Not reported
Ornstein M.H., 1981 [36]	T1: Bran (6.99 gr/day) T2: Ispaghula (9.04 gr/day) T3: Placebo (2.34 gr/die)	4	Reduction of abdominal symptoms	No change in pain. T1 and T2: improvement of straining (*p* < 0.01)	T2: 2 pts diarrhea T3: 1 pt constipation
Eastwood M.A., 1978 [37]	T1: Bran (20 gr/die) T2: Ispaghula (2 sachets/day) T3: Lactuose (20–40 mL/day)	1	Reduction of abdominal symptoms	T1, T2 and T3 alleviated symptoms	Not reported
Hodgson W.J.B., 1977 [38]	T1: Methylcellulose 500 mg bid T2: Placebo	3	Reduction of abdominal symptoms	Symptoms score decrease significantly in T1 (from 19 ± 6, to 13 ± 4 *p* < 0.01) but not in T2 (from 21 ± 7 to 17 ± 9, *p* = ns)	Not reported
Brodribb A.J.M., 1976 [39]	T1:Wheat bran 24 gr/die	6	Reduction of abdominal symptoms	60% of symptoms were abolished and 28% relieved	Not reported
Taylor I., 1976 [40]	T1: High roughage diet with bran supplement T2: Bulk laxative plus antispasmodic T3: Bran tablets (18 gr/day)	2	Reduction of abdominal symptoms	T3 was most effective in reduce symptoms. Asymptomatics pts were: T1 = 20%; T2 = 40%; T3 = 60%	Not reported

FU: follow-up; pts: patients; T1: treatment arm 1; T2: treatment arm 2; T3: treatment arm 3.

## Supplementary Materials

The following are available online at http://www.mdpi.com/2072-6643/9/2/161/s1.
