# Supplementary material for: Role of Fiber in Symptomatic Uncomplicated Diverticular Disease: A Systematic Review
Source: Nutrients. 2017 Feb 20;9(2):161. doi: 10.3390/nu9020161 (PMC5331592; doi:10.3390/nu9020161)
Supplement: Supplementary file 1 [file nutrients-09-00161-s001.doc]

**Supplementary Table S1. Quality assessment according to Jadad scale**

| **Author, year** | **1)** | **2)** | **3)** | **4)** | **5)** | **6)** | **Score** |
| --- | --- | --- | --- | --- | --- | --- | --- |
| **Dietary** |  |  |  |  |  |  |  |
| Lahner E, 2012 | 1 | 0 | 1 | 1 | 0 | 0 | **3** |
| Annibale B, 2011 | 1 | 0 | 1 | 1 | 0 | 0 | **3** |
| Colecchia A, 2007 | 1 | 0 | 1 | 1 | 0 | 0 | **3** |
| Smits BJ, 1990 | 1 | 0 | 1 | 0 | 0 | -1 | **1** |
| Leahy AL, 1985 | 0 | 0 | 0 | 0 | 0 | 0 | **0** |
| Hyland JMP, 1980 | 0 | 0 | 0 | 0 | 0 | 0 | **0** |
| Brodribb AJM, 1977 | 1 | 1 | 0 | 0 | 1 | -1 | **2** |
| Plumley PF, 1973 | 0 | 0 | 1 | 0 | 0 | 0 | **1** |
| Painter NS, 1972 | 0 | 0 | 1 | 0 | 0 | 0 | **1** |
| **Supplementary** |  |  |  |  |  |  |  |
| Lanas A, 2013 | 1 | 0 | 1 | 1 | 0 | 0 | **3** |
| Latella G, 2003 | 1 | 0 | 1 | 1 | 0 | 0 | **3** |
| Papi C, 1995 | 1 | 1 | 1 | 1 | 1 | 0 | **5** |
| Papi C, 1992 | 1 | 0 | 1 | 1 | 0 | 0 | **3** |
| Thorburn HA, 1992 | 0 | 0 | 0 | 0 | 0 | 0 | **0** |
| Ornstein MH, 1981 | 1 | 1 | 1 | 0 | 1 | -1 | **3** |
| Eastwood MA, 1978 | 0 | 0 | 0 | 0 | 0 | 0 | **0** |
| Hodgson WJB, 1977 | 1 | 1 | 0 | 0 | 1 | -1 | **2** |
| Brodribb AJM, 1976 | 0 | 0 | 0 | 0 | 0 | 0 | **0** |
| Taylor I, 1976 | 1 | 0 | 0 | 0 | 0 | -1 | **0** |

1=yes; 0=no;

1) Was the study described as randomized?

2) Was the outcome assessment described as blinded?

3) Was there a description of withdrawals and dropouts?

4) Was the method of randomization well-described and appropriate?

5) Was the method of blinding of the assessment of outcomes well-described and appropriate?

6) Deduction of 1 point if methods for randomization or blinding were inappropriate.
